# UVR Exposure and Prevention of Street Construction Workers in Colombia and Germany

**DOI:** 10.3390/ijerph19127259

**Published:** 2022-06-14

**Authors:** Mayra F. Calvache Ruales, Stephan Westerhausen, Hernan A. Zapata Gallo, Benjamin Strehl, Sergio D. Naza Guzman, Helmut Versteeg, Wiho Stöppelmann, Marc Wittlich

**Affiliations:** 1 Risk Management Consultancy CGR, SURA, Cali 760046, Colombia; mcalvache@sura.com.co (M.F.C.R.); snaza@sura.com.co (S.D.N.G.); 2 Department of Ergonomics: Physical Environmental Factors, Institute for Occupational Safety and Health of the German Social Accident Insurance, D-53757 Sankt Augustin, Germany; stephan.westerhausen@dguv.de; 3 Occupational Risk Insurance ARL, SURA, Medellin 050034, Colombia; hzapatag@sura.com.co; 4 Department Accident Prevention: Digitalisation—Technologies, Institute for Occupational Safety and Health of the German Social Accident Insurance, D-53757 Sankt Augustin, Germany; benjamin.strehl@dguv.de; 5 Department Exposure and Risk Assessment, Institute for Occupational Safety and Health of the German Social Accident Insurance, D-53757 Sankt Augustin, Germany; helmut.versteeg@dguv.de (H.V.); wiho.stoeppelmann@dguv.de (W.S.)

**Keywords:** ultraviolet radiation, personal dosimetry, risk assessment, occupational health, outdoor work, occupational health prevention

## Abstract

(1) Solar ultraviolet radiation (UVR) poses a major risk factor for developing skin cancer after years of chronic exposure. The irradiation is strongly dependent upon the activity or occupation carried out, but also on the climate conditions at the workplace. Knowledge of both has been tested within the occupational group of road construction workers in Colombia and Germany. (2) The GENESIS-UV measurement system has been used at both locations for consistency. A number of workers in both countries wore an electronic data logging dosimeter for several months to deliver detailed information on UVR exposure. (3) It was found that in a tropical climate, UVR exposure remains constant throughout the year, while in a temperate climate seasonal effects are visible, superimposed by behavioural aspects e.g., in springtime. The daily distribution of the radiation shows a distinct dip, especially in the Colombian data. Derived data show the high fraction of working days exceeding a threshold set by the skin type. (4) Road construction work involves high UVR exposure. In both countries, preventive measures are required to reduce the personal exposure to a minimum. Exceedance of the minimal erythema dose (MED) suggests a possible enhancing effect, especially in fair skinned people. Intercomparison of UVR exposure at workplaces is possible between countries and climate zones, emphasizing efforts for global action against skin cancer.

## 1. Introduction

Humans are constantly exposed to environmental influences in their lives. These can be of various natures and act in different ways. The interaction of optical radiation with the human body can lead to risks to health. Although the eyes and skin, as the primary receptors of radiation, are equipped with mechanisms to prevent or repair damage, this is highly dependent on the irradiation (the dose) [1]. At a certain point, the repair mechanisms are no longer able to prevent sustainable damage.

Skin cancer is a malignant change and the uncontrolled growth of skin cells. Although melanocytic and non-melanocytic skin cancers (NMSC) involve different cell types, the molecular mechanisms in the development are comparable if ultraviolet radiation (UVR) is the cause [2,3]. The incident UV photons cause direct damage to the DNA. Whether or not skin cancer develops depends on many factors, including genetic predisposition and environmental and nutritional factors [4,5,6]. Therefore, it is difficult to conclude the probability of later disease based on phenotypical characteristics alone. Human skin has been classified according to its sensitivity to solar UVR on a six-stage Fitzpatrick scale that is used today as a standardised basis for skin cancer risk assessment [7].

In the case of NMSC in particular, a distinction is made between different skin cancer types which, although caused by UVR, are likely to be subject to different patterns of exposure in the process [8].

Squamous cell carcinoma (SCC) is a classic example of a tumor that is caused by cumulative sun exposure, and is in this case linked to areas with high exposure to UVR. SCC have precursors in their development, the actinic keratosis (AK). The prevalence of actinic keratosis in Germany was stated to be 11.5% for 60 to 70 year olds [9]. A Dutch study found a prevalence of 28% in women and 49% in men in a study population with an average age of 72 years [10]. Basal cell carcinoma (BCC) is about four times more common than SCC and is the most common skin cancer worldwide in the white age group between 40 and 79 years [11,12,13].

Based on the available information from six cancer registries, it is estimated that about 213,000 people in Germany develop NMSC each year, of which about 77% have BCC, and 22% have SCC, with other entities accounting for 1%.

The incidence of both SCC and BCC is increasing worldwide [14,15,16,17,18], but skin cancer rates are decreasing in Australia and New Zealand, at least for melanoma [19,20,21], where decades of prevention work have succeeded in reducing new cases among young adults.

The measurement of UVR exposure has a long tradition. In recent years, however, a decisive development has taken place, especially in the field of measuring technology for devices worn by people. While in the past only simple polysulphone film dosimeters were used (see e.g., [22,23,24,25,26,27]), today electronic data logger dosimeters are increasingly used [28,29,30,31,32,33,34]. The advantages and disadvantages of the different dosimeter types have been previously reported, and these were considered in the selection of monitoring instruments used in this study [33].

Almost all studies in occupational settings provide cumulative results for occupational groups, such as construction workers, without going into the resolution of individual occupational profiles. Even rarer is a seasonal breakdown or documentation of the irradiation of individual subjects of an occupational group. Earlier work, for example, dealt with the exposure of pilots or other flight personnel [35], the exposure of schoolchildren [36], or sports teachers [37,38,39]. High UV exposures have been reported in the horticulture and agricultural sectors [40,41,42].

The construction sector has always been associated with high irradiation. However, it is problematic that even in the construction sector a wide variety of activities are carried out. A generalization is therefore not possible and must always be made on an occupational basis. Information on exposures in the construction industry has been published; in addition to the general term of construction worker, this also includes occupations such as carpenter and brick layer [24,43,44,45].

Two studies explicitly mention road construction workers. A study from Poland and a study from New Zealand find high daily irradiation levels of 11.5 SED in July and 5.3 SED in January, respectively, measured at or on the shoulder [44,45].

Our study focuses on two questions. First, we aimed to obtain detailed exposure data for the occupational group of road construction workers in both countries. One focus was to be able to perform a statistically reliable exposure measurement through a high number of test persons. Furthermore, the level of detail should be high enough to allow a statement about the course of exposure over the year and over the day. Another question was whether there are significant differences in the annual course of exposure between a country in the tropics and in the temperate zone, and if so, whether these are so great that they can influence prevention concepts.

To our knowledge, there has been no study in the field of occupational health and safety in connection with UVR exposure measurements that pays attention to the specifics of the earth’s climatic zones. The tropical climate is subject to diurnal variation, whereas the climate in the temperate zone has seasons. This is due to the inclination of solar radiation, which is also directly linked to UVR intensity.

## 2. Materials and Methods

The same technique was used at both measurement sites. From this we conclude that any influences by the measuring system could occur equally, and thus cancels out when comparing findings at the two sites. However, we minimised such an influence through the design of the measuring system.

Air pollution can basically influence the UV irradiation level. Primarily, it is assumed that there is a reduction in UV-B irradiation. Secondary effects such as back reflection or changes in behaviour (reduced wearing of protective clothing due to increasing heat and humidity) can, however, produce an opposite effect. Aspects of air pollution are, however, negligible in this study, as it concerns long-term measurements at various construction sites. These measurements took place in different regions of Germany and Colombia and included non-urban areas in particular.

No intervention took place before the measurements, so everyday behaviour can be assumed given the long duration of the measurement campaign. Any Hawthorne effect, if present, is therefore negligible.

### 2.1. Participants and Locations

#### 2.1.1. Colombian Subset

In Colombia, six road construction workers were voluntarily recruited in 2017 and 2018, and they wore dosimeters every working day for the entire shift over a period of eight months (August to March). The measurement time was from 7:30 a.m. to 5:30 p.m. UTC-5. The workers were distributed in three regions of the country. The main activities of the workers were the construction of civil engineering works, i.e., public roads, bridges, viaducts, road maintenance, and traffic regulation.

**Figure 1:**
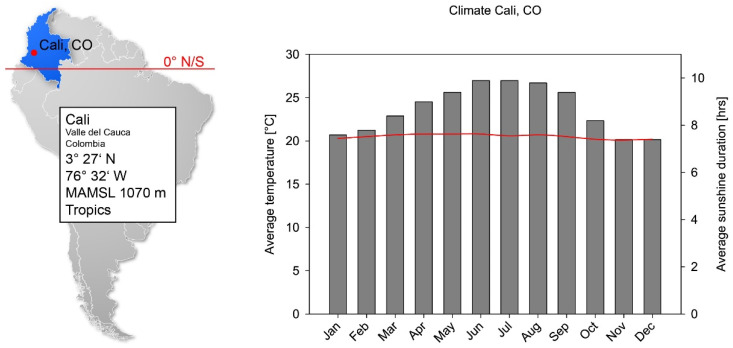
Geographic and climate conditions of Cali, Colombia. Position and meters above main sea level (MAMSL) are given. The temperature only varies on a decend scale (grey bars), and the average sunshine remains virtually constant throughout the year (red line). The acceptance of prevention measures depends on the first and daily irradiation on the second parameter.

Cali was defined as the reference location (see Figure 1). Although Colombia has different altitude levels, the measurements were taken below 1000 m.

The workers involved in the study were affiliated with an occupational risk insurer in Colombia (SURA). The main criterion for the execution of the project was to have a working population in positions with tasks and outdoor activities for most (greater than 70%) of their working day. The companies that allowed the project to be undertaken were visited, and the criteria to be met were evaluated. The measurements were made on the people chosen for their work activity within the engaged companies.

The participants from Colombia were on average 30.3 ± 3.5 years old, with the youngest participant being 25 years old and the oldest being 40 years old.

#### 2.1.2. German Subset

In Germany, 31 road construction workers were voluntarily recruited in 2014 and 2015, and all wore dosimeters every working day for the entire shift over a period of seven months (April to October). The measurement time was therefore pre-set to 7:30 a.m. to 5:30 p.m. CEST. The workers were distributed all over the country, but carried out activities similar to their colleagues in Colombia. These included setting up/clearing construction sites, earthworks, constructing road drainage facilities, producing frost, base and surface courses, paving verges, as well as repair and maintenance work.

**Figure 2:**
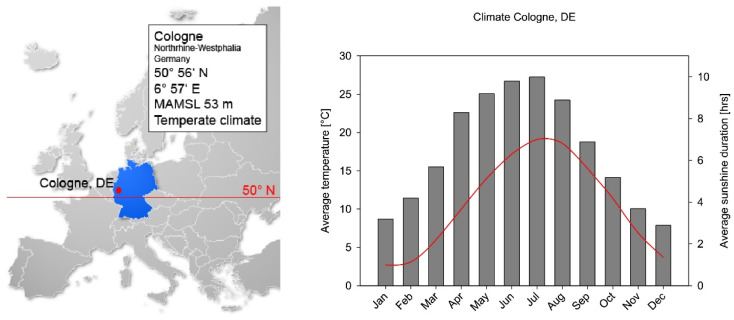
Geographic and climate conditions of Cologne, Germany. Position and meters above main sea level (MAMSL) are given. The temperature varies strongly throughout the year (grey bars), and the average sunshine follows a comparable course (red line). The acceptance of prevention measures depends on the first and daily irradiation on the second parameter.

During the evaluation of the data, the exact measurement location was taken into account; for the sake of simplicity of presentation in this paper, Cologne is defined as the reference location (see Figure 2). Germany only has a few locations that are situated above 1000 m above sea level, and none of the measurements were carried out there. Therefore, it can be concluded that all measurements took place at the same altitude level, which was not influenced by mountainous locations.

For participation, companies were approached by the employer’s statutory sical accident insurance institution for the building trade who could provide volunteers for the measurement campaign. Each volunteer could terminate participation at any time without reason. The participants from Germany were on average 39.6 ± 12.9 years old, with the youngest participant being 17 years old and the oldest being 65 years old. We conclude that we were able to cover every stage of development within the profession.

### 2.2. Measurement Technology

The GENESIS-UV (GENeration and Extraction System for Individual expoSure) measurement system was used to measure workers’ exposure to UVR. This is a system specifically designed for decentralised long-term measurements, which in particular allows the use of electronic data logger dosimeters in a wide variety of locations around the world. Studies using the measurement system have been published elsewhere [34,46,47,48,49,50].

Each test person was equipped with a complete unit of the measuring system. The measuring device is an electronic data logger dosimeter X2012-10 (Gigahertz, Türkenfeld, Germany), which is worn on the left upper arm. This dosimeter records UVR in the UV-A and UV-B/C channels at one-second intervals. The incident radiation is weighted by filter combinations directly according to the erythema action spectrum S_er_(λ) [51]. It has become common practice in the field to assess measurements with regard to solar UVR exposure with the erythema action spectrum, although there is also one with NMSC weighting. The latter, however, is widely rejected by medical experts because it was determined in albino mice and the results are not transferable to humans. Furthermore, the dosimeter contains a three-axis accelerometer, as well as a three-axis magnetometer (Bosch eCompass BMC-150, Bosch, Stuttgart, Germany), which recorded movement and magnetic field direction. In this configuration, the internal memory of the dosimeter has a recording capacity of 54 h. The dosimeter is then connected to the tablet PC belonging to each GENESIS unit. The readout process and the data transfer via mobile data service or WLAN are started automatically by the GENESIS client. After the data readout has been completed and a local data backup has been saved, the dosimeter memory is automatically cleared, the battery is charged and the unit is restarted.

By using the acceleration sensor in the dosimeter, a direct analysis of the data regarding wearing by test persons is possible.

**Figure 3:**
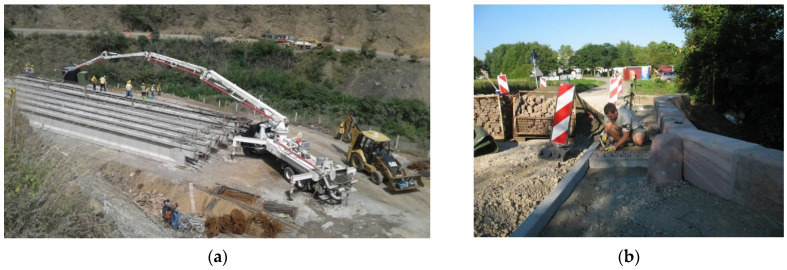
Photographs of construction sites and workers in Colombia while working on bridge construction (**a**) and in Germany while paving a walkway (**b**). The dosimeter is worn on the upper left arm (black strip) without any impairment of the task being carried out. (©SURA, ©IFA).

Photographs of the construction sites and the wearing position of the dosimeters are shown in Figure 3.

### 2.3. Data Analysis and Extrapolation

The aim of processing the measurement data is to turn the raw data into measurement values for scientific evaluation. This requires several steps, including the creation of metadata records, as individual pieces of information recorded or stored independently of each other have to be linked together. The data read out from the dosimeter for irradiation, acceleration and magnetometer data are first calibrated to the measurement location and time. Furthermore, any corrections due to technical ageing processes of the dosimeters are taken into account.

The accelerometer data are also calibrated according to the manufacturer’s specifications and combined into a vector amount. The aim is to determine the deviation from the uniform, exclusively earth-accelerated movement. This serves as the basis for determining the activity of the test persons and thus as the basis for the quality evaluation of the data.

In the next step, the channel-by-channel calibrated irradiation is combined into a sum to be able to determine the total irradiation in the UV range. This is done on the basis of the second values, which are then used to form further metadata (half-hourly values and daily values, for example). Both the daily totals and the half-hourly values are arithmetically averaged on a monthly basis. For this purpose, the sum of the valid individual values processed according to the data reduction is formed and then divided by the number of measured values. For averaging the half-hourly values, the respective half-hourly values of the individual valid days are summed and then divided by the number of values used.

At the end of the data processing, exposure values for whole days as well as the half-hourly values are available. This makes an evaluation at the occupational and sub-occupational level possible.

Assumptions have to be made for the further development of values spanning a larger period of time. This means that from the extrapolation of daily averages to monthly totals, it must be determined how many working days per month are to be applied.

## 3. Results

In both countries, a sufficient amount of data could be collected to be able to calculate statistically validated mean values. The monthly daily averages for both countries are shown in Table 1, a graphical representation as well as an analysis of the data is first done country by country. Even at first glance, it can be seen that there are considerable differences both in the absolute level of exposure and in the course of the year.

### 3.1. Tropical Zone: Colombia

**Figure 4:**
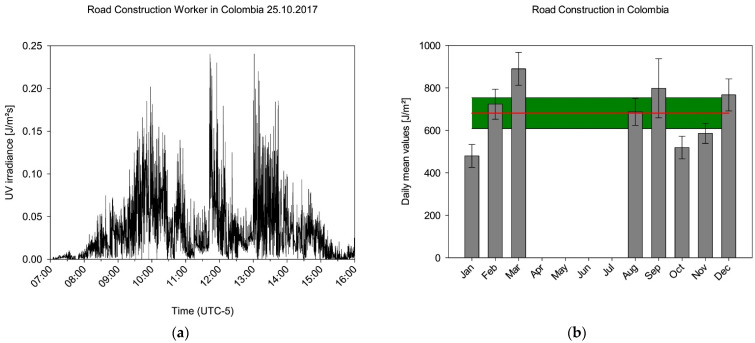
(**a**) Example of a data stream from a participant in Colombia on 25 October 2017. Episodes of different exposures from the sun can be identified. (**b**) Daily mean values for street construction workers in Colombia. From April to July, no data were acquired. As Colombia is a country with a daytime climate, a mean daily irradiation could be calculated by combining all data available (red line, standard error coloured green).

The measurements carried out in 2017 yielded 205 measurement days of the highest category in Colombia, together with 4866 half-hourly values of the highest category. Figure 4 shows an example of a complete measurement day of the highest category of a road construction worker in Colombia. The course of the day, which is typical for the tropics and is characterised by a rather rapid sunrise, can be recognised in the profile. In addition to episodes in direct irradiation, there are phases that were apparently carried out in partial shadowing (approx. 11:30 a.m. and 12:30 p.m.).

The measured values could be included in the analysis on a monthly basis and allowed the formation of monthly daily averages (Figure 4). It can be clearly seen that there is no seasonal progression. The individual mean values are subject to fluctuations, which will be discussed later on, but which agree well within the framework of their error observation. If we now assume in a further step that all data are not subject to a season independently of each other, then they can also be evaluated together. If one forms the mean value from the complete data set, then a daily mean value of 681 J/m^2^ ± 73 J/m^2^ (red line, Figure 4) valid for all days of the year results.

**Figure 5:**
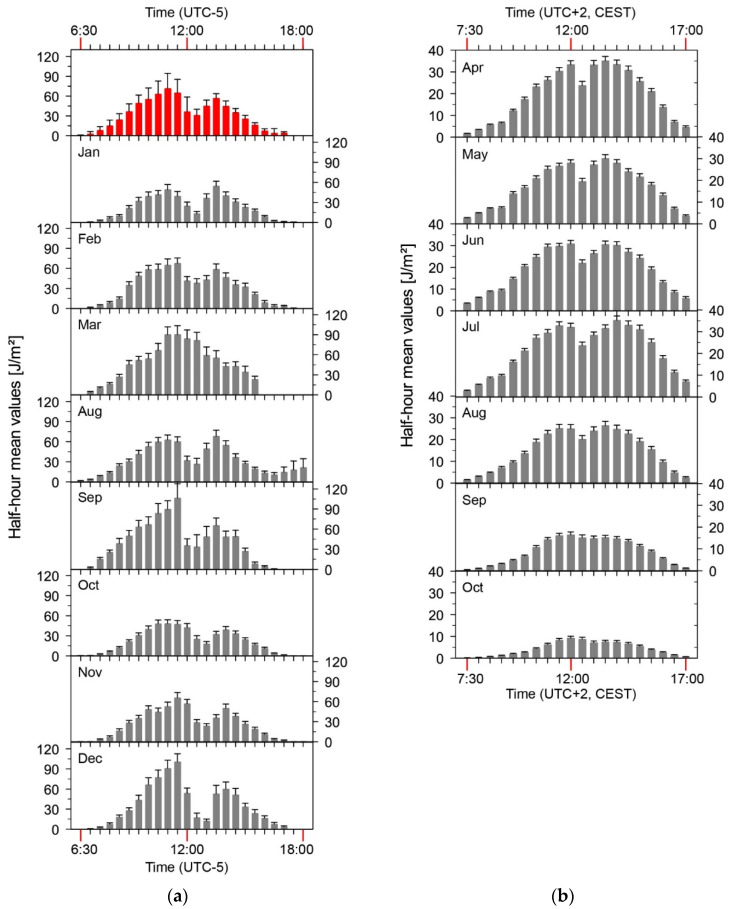
Half-hour mean values for street construction workers. (**a**) Colombia. A mean of all half hours is shown on top (red, with standard deviation), and time is local standard time (UTC-5). The daily maximum occurs around 12:00 p.m. (**b**) Germany. The time is Central European Summer Time (UTC+2, CEST). The daily maximum occurs around 1:00 p.m. For every month, mean half hours are calculated with their standard error.

A similar picture can be derived by looking at the half-hourly values (Figure 5a). The diurnal course seems to be comparable over the months, also with regard to the maxima and minima. Exposure is highest before and after a supposed lunch break. The break, which is presumably spent in the shade, causes a reduction in exposure of about 50%. The periods of voluntary deprivation of sun exposure at noon in tropical countries is common due to a natural adaptive process. Reduced activities at midday are undertaken to protect from high UVR exposure, depicted by a high ultraviolet index (UVI). Culturally, it is associated with long periods of rest and feeding compared to those countries where there are seasonal variations in solar exposure.

### 3.2. Temperate Climate: Germany

**Figure 6:**
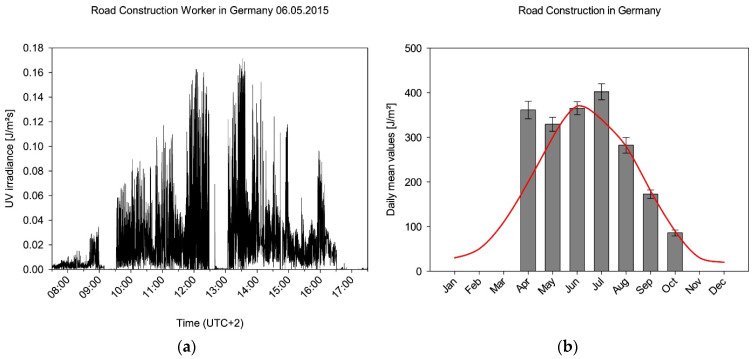
(**a**) Example of a data stream from a participant in Germany on 6 May 2015. Episodes of different exposure against the sun can be identified. (**b**) Daily mean values for street construction workers in Germany. From November to March, no data have been acquired. As Germany lies within a temperate climate, the solar UV irradiance varies throughout the year (red line, factors from [52]). The red curve is normalised to the maximum of global UV irradiance in June.

In total, participants in Germany collected 2095 complete measurement days of the highest category, as well as a total of 45,914 half-hourly values. Figure 6a shows a typical profile of a measurement. It can be clearly seen that the test person spends a break in the morning and at noon in the sun-protected area. Furthermore, the typical course of the UVR intensity by the sun during the day can be taken from the representation.

All measurement data are used in the further evaluation and derivation of cumulative values as described. Figure 6b shows the monthly daily mean values for road construction workers in Germany, and those values are also listed in Table 1.

The data determined in the months from April to October qualitatively follow the expected course, which results from the seasonal course of the sun’s global irradiation. One can roughly deduce that the irradiation between October and June differs by a factor of more than four. Obviously, however, the exposure in April deviates significantly from this course, which can be explained by human behaviour in spring. People deliberately expose themselves to the sun’s rays in spring to absorb warmth when the air is still cool and to feel the sunshine as a feel-good factor.

If we look at the average half-hourly values for the individual months, the qualitative course of the UV irradiation intensity is also imminent there (Figure 5b). The basic course is the same in each month, no exposure episodes on days that have a particularly high or a particularly low exposure compared to the expected theoretical distribution are apparent.

In all months, however, the supposed midday break from 12:30 p.m. can be identified, which is associated with a reduction in exposure of up to 25%. If an envelope were placed over the individual graphs, there would be good agreement with the theoretical daily distribution of UV irradiation, which has its maximum at around 13:00 CEST.

### 3.3. Derived Findings

The determination of UV irradiation reveals clear differences in the comparison between Colombia and Germany. While in Colombia the daily irradiation maxima—derived from the half-hourly values—can be as high as approx. 90 J/m^2^, in Germany this is about 35 J/m^2^. This is associated with a factor of about 2.6.

The difference is somewhat less pronounced when looking at the monthly daily mean values. Between the month with the highest daily mean value in Colombia (March, 890 J/m^2^) and the month with the highest daily mean value in Germany (July, 402 J/m^2^), a factor of 2.2 is found; if we take the global mean value of the data from Colombia (681 J/m^2^) as a reference, a factor of only 1.7 can be derived.

For the extrapolation to the annual irradiation, the seasonal or diurnal distribution of the global solar UV irradiation gains weight. Whereas in Colombia there is a high level of UV irradiation all year round, in Germany this is subject to significant change. There is a factor of 13 between the irradiation in December and June (Table 2). This is particularly significant when extrapolating to an annual exposure. If 20 working days are assumed for the months of April, June and September, and 21 working days for the months of May, July, August and October, then a total amount of irradiation can be calculated for the measurement period. If one also takes into account via the seasonal factors that the irradiation in the measurement period corresponds to 88% of the total annual irradiation, then one can extrapolate to the entire year. For road construction workers in Germany, this results in an annual irradiation of 46,662 J/m^2^ or 466 SED (1 SED = 100 J/m^2^ erythema-weighted irradiation; wavelength spectrum evaluated according to erythema effectiveness according to CIE [51]). In comparison, 230 working shifts per year are assumed for the road builders in Colombia, plus the average value per day of 681 J/m^2^. This results in an annual irradiation of 156,630 J/m^2^ or 1566 SED. Thus, a factor of 3.4 in the annual irradiation between Germany and Colombia can be elucidated, determined from exposure measurements with uniform technology.

**Table 2:** Top: Prevalence Quota for Colombia (CO) and Germany (DE), also illustrated in Figure 7. The ratio indicates how many times higher the exceedance is in Colombia. Center: Taking the level of 3 SED as an example, it is indicated how the exceeding of the levels in Germany is distributed on a monthly basis. Bottom: Seasonal factors for Germany describing the proportion of the yearly irradiation for each month [52]. The single values sum up to 1.0 for the whole year.

Overall Quota of Values above Level Indicated												
		>1 SED		>2 SED		>3 SED		>4 SED		>6 SED		>9 SED
**CO**		0.95		0.89		0.79		0.68		0.49		0.24
**DE**		0.74		0.54		0.38		0.26		0.11		0.03
**Ratio CO/DE**		1.28		1.64		2.08		2.61		4.40		7.14
**Monthly Quota of Values > 3 SED of Road Construction Workers in Germany**												
**April**		**May**		**June**		**July**		**August**		**September**		**October**
0.48		0.46		0.51		0.57		0.37		0.18		0.04
**Seasonal Factor for Latitudes Like Germany**												
**Jan**	**Feb**	**Mar**	**Apr**	**May**	**Jun**	**Jul**	**Aug**	**Sep**	**Oct**	**Nov**	**Dez**	**Σ**
0.015	0.025	0.055	0.100	0.150	0.185	0.170	0.140	0.090	0.045	0.015	0.010	1.0

**Figure 7:**
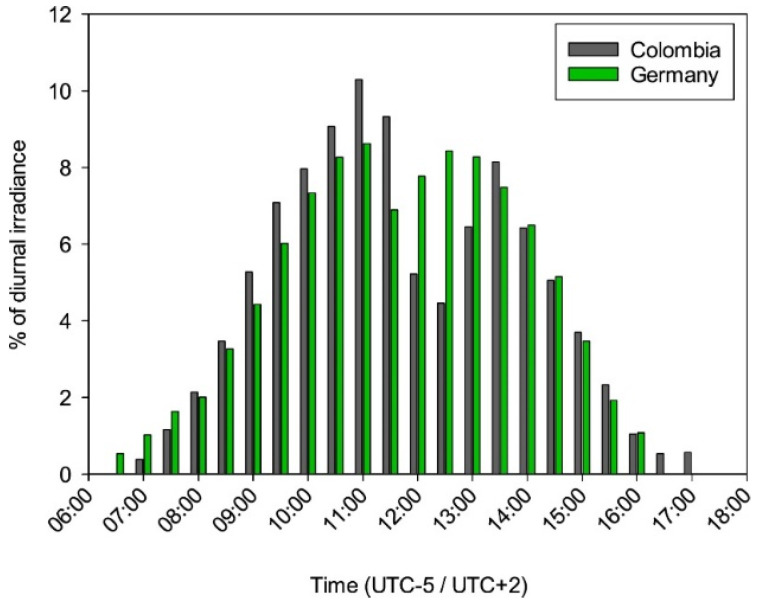
Proportion of irradiation in half an hour of the total daily irradiation for Colombia (grey, UTC-5) and Germany (green, UTC+2). The basic values for the calculation from the irradiation for Colombia are equal to the values of the top graph in Figure 5; for Germany a mean irradiation value per half hour was formed as an average of all months.

**Figure 8:**
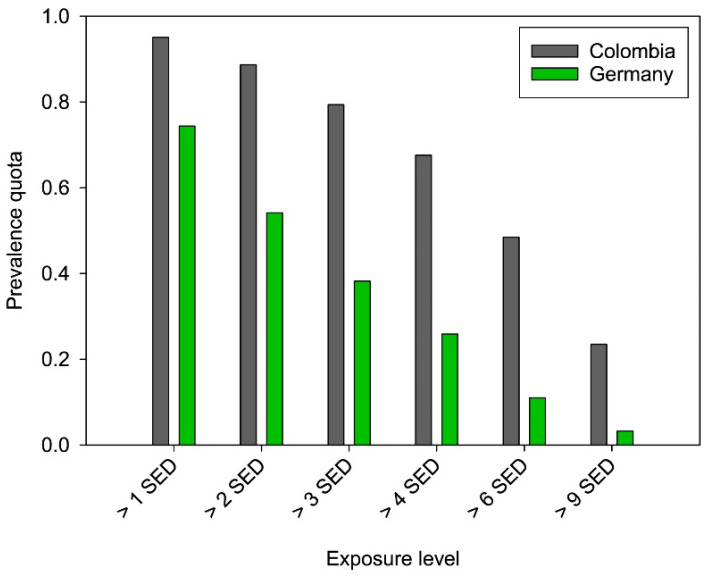
Prevalence Quota depicting the number of values above an exposure level in relation to the total number of values for Colombia (grey) and Germany (green). The exposure levels are related to the Minimal Erythemal Dose (MED) for skin types following the Fitzpatrick scale [7].

Even more information can be derived from the individual measurement days. If one looks at the number of days above certain exposure levels, then one can calculate in relation to the number of total measured values how high the prevalence is for exceeding limit values. If one knows the actual minimum erythema dose (MED) according to Fitzpatrick, then even statements about risk levels can be derived. Figure 8 illustrates these prevalence rates for Colombia and Germany. As expected, the number of risk-exceeding days in Colombia is at all times higher than in Germany, and the ratio between the countries even continues to rise. At a level of >1 SED it is 1.3, while at >9 SED it is 7.1 (Table 2). Looking at the absolute values, at a threshold of >9 SED, about 24% of the values in Colombia are still above the level, while in Germany it is still about 3%.

Surprisingly, the skin type distributions according to Fitzpatrick for the majority of citizens in both countries are comparable. In the German population, skin type is distributed as 4% skin type I, 57% skin type II, 35% skin type III and 4% skin type IV. Skin types V and VI are only represented in a very small percentage. Similar figures are reported from Colombia [53].

The majority of the Colombian population has skin type II-III according to the Fitzpatrick scale [54]. The MED for skin type III is in the range of 3 SED to 5 SED. If 3 SED is taken as a basis, then for road construction workers in Germany about 38% of the daily exposure values are above the level, in Colombia about 79% are above it. While in Colombia these are distributed over the whole year, maxima in Germany can be detected in summer (June/July, Table 2). In these months, more than 50% of the measured values are above 3 SED, but in April and May they are insignificantly below this level. On the basis of these findings, whether the level of exceedance of the risk level is tolerable can be determined; in other words, if a level has been specified, whether it is adhered to in the road construction profession can be calculated.

### 3.4. Detailed Analysis of the German Data

In Germany, the participants were asked to keep a diary of the activities they carried out. The list of activities was agreed upon and specified by experts beforehand so that all test persons could ultimately provide the same information. The fields of work of the German participants were the same as those of the Colombians (see the Materials and Methods section of this paper), with individual activities such as construction of road drainage facilities, construction of frost, base and surface layers, construction of edge stabilization, as well as setting up/clearing and securing the construction site, among others. Each activity is associated with a certain irradiation, which indicates how high the risk is and what proportion of the total irradiation in the occupation this activity can have. By identifying individual activities associated with high exposure, protective measures can be specifically applied there.

In order to be able to compare the risk of the individual activities, the irradiation is calculated as if the activity were carried out exclusively for the entire year. A comparison can then be made on the basis of the annual exposure values.

In the case of the activities for the road construction workers, it was found that that repair and maintenance work was associated with the highest annual exposure (802 SED), followed by the production of edge fixings (605 SED). The construction of frost, base and wearing course (547 SED) was ahead of demolition and caulking (483 SED) and road drainage (435 SED). Earthworks (419 SED) and site installation/clearing and securing (385 SED) rounded off the picture. The focus of each worker’s activities then leads to the actual annual exposure of a road construction worker. The differences between the individuals then results in the dispersion within the occupational group.

## 4. Discussion

### 4.1. General Findings

To the best of our knowledge, our project is the first comparative study between occupational UVR exposures of workers in different climates. In order to reduce technical imponderables to a minimum, we used identical measurement technology in both countries. The reliability of the GENESIS-UV measurement system has already been demonstrated in several studies, as stated above. Measurements of UV irradiation are a major challenge, both with regard to technical-logistical aspects and the choice of the right approach. This includes, for example, questions about the duration of measurement campaigns, as well as the recruitment of a sufficient number of subjects, but also the consideration of climatic conditions and their seasonal changes. The measurement of exposure took place on clothing and therefore cannot take this into account. Due to the many additional questions about clothing (thickness, length, weave, etc.), a reproducible measurement would be impossible. The influence of clothing or sunscreen, for example, would therefore have to be investigated in another study.

When recruiting test persons in both countries, care was taken to ensure that they were employed customary for the job. Accordingly, it can be concluded that the observed differences in irradiances can primarily be attributed to the respective climatic conditions. Ultimately, the sun is the determining factor for global irradiation, which is also reflected in the different levels of the UV index.

Colombia is a country that follows a diurnal climate, whereas Germany follows a seasonal climate. This is not only shown by the climatic parameters (Figure 1 and Figure 2), but also clearly in the distribution of UV irradiation over the year (Figure 4b and Figure 6b). This difference is reflected in the monthly daily mean values by a factor of 2.2 and 1.7, respectively, with which the irradiation in Colombia is higher than in Germany. If one applied the latitude factor to these monthly values as well, one would have expected a factor of 3.8 [52]. Due to the different seasonal patterns, the extrapolation to the year is clearly different, so that a factor of 3.4 results from the ratio of the measured irradiations in Colombia and Germany. This factor, determined from our measurements, has a certain sensitivity to the number of working days used for extrapolation. If one deviates from the 230 working days used for the calculation in Colombia by only ten days upwards or downwards, then a factor of 3.5 or 3.2 results. An estimation of the sensitivity of the value determined in Germany to the number of working days is somewhat more difficult, since the extrapolation to the full year could not be carried out using measured values from November to March. However, since only a total of 12% of the annual irradiation occurs in these months, the associated error is small. The highest irradiances also occur at the time of the sun’s peak in June and the subsequent summer, so that the influence of the number of working days in these months on the annual irradiance is more significant. The extrapolation would change if individual holidays had to be taken into account in these months. This would mean that the annual irradiation of the majority of the employees would be lower with the effect that the ratio to the irradiation of an employee in Colombia would increase further. All of these aspects mean that our measurements are in good agreement with the theoretical consideration of the latitude factor of 3.8.

The monthly daily mean values in Table 1 indicate how high the daily irradiation is on average in the particular month. This average value naturally includes both higher and lower individual measured values. It is therefore quite possible that employees are irradiated with even higher daily doses. Wolska et al. (2013) state that road construction workers in Poland receive 11.5 SED on the shoulder in July [45]. Taking into account the body site factor between the left upper arm and the shoulder of 1.5 [24], the data obtained in Germany would result in an irradiation of 6 SED.

However, if one takes into account that the measurements in Poland were made on and not at the shoulder, then a body site factor comparable to the top of the head would have to be used. This value of 3.3 would result in an irradiation in July of about 13 SED, which shows good agreement of both measurements.

Another possibility for comparison is a measurement by Hammond et al. (2009) from New Zealand [44]. These measurements took place on the posterior clavicle (shoulder blade) and must therefore be transformed with the factor for the shoulder of 1.5. The 6 SED we measured agree very well with 5.3 SED measured in New Zealand. Both the measurement site in Poland and the measurement site in New Zealand are at the same latitude level as Germany, so conversion using the latitude factor was not necessary.

With regard to the total irradiation per year, the measurements in Germany and Colombia corresponded very well. In conclusion, the measurements in Colombia are in good agreement with those in Poland and New Zealand.

The daily distribution of UV irradiation differs between Germany and Colombia in some important aspects. While the basic course of the day is similar, a significantly reduced exposure can be observed, especially around midday in Colombia. This can be explained by the fact that in Colombia, in contrast to Germany, an extended break in connection with meals is customary. This offers great preventive potential, which should be taken into account when choosing protection measures. A lot of irradiation is acquired in a short time, especially around the time of the sun’s peak. According to the distribution of UV irradiation in Germany on a summer day, this is 66% between 10:00 a.m. and 3:00 p.m. For Colombia, this proportion is somewhat larger, as the radiation maximum is more sharply concentrated around midday. If one looks from the theoretical consideration to the actual distribution of irradiation on a day, distributions emerge in which the behavior of the employees is also reflected to a certain extent. Figure 7 shows the percentage share of the mean half-hour value in the total irradiation distribution of the day for both countries. In comparison, it is directly noticeable that especially around 11:00 a.m., significantly higher percentages are collected in Colombia than in Germany, while the reverse is true at approximately 1:00 p.m. If we look at the period between 10:00 a.m. and 3:00 p.m., which is relevant for occupational health rules in Germany [55,56], then on average about 75% of the daytime exposure is collected in this period in Germany, and about 72% in Colombia.

Both proportions clearly show that the preventive potential for the period when the sun is at its highest is far from exhausted and that organizational measures in occupational health and safety (e.g., relocation of activities outside the time of peak sunshine) could be helpful.

From our measurements, it is basically possible to deduce how high the irradiation of the individual employee is at a certain point in time. The measurement data inherently provide information about the effect of already introduced technical and organizational preventive measures in combination with behavioural preventive measures. Personal protective measures such as clothing cannot be taken into account, as the measurement takes place on the clothing. Even if large areas of the body such as arms, legs or the surface of the head are covered by clothing, many areas of the skin such as the face or hands are usually uncovered. In these places, the measured values become particularly relevant, possibly even more so than indicated, if the body part in question is oriented more perpendicular to the sun than the upper arm used for the measurement. The importance of reducing the daily irradiation as much as possible becomes particularly obvious when looking at the rate of days exceeding the limit value (Figure 8). Especially for fair skin types, the rate is so high that permanent and, in the long run, chronic skin damage is to be expected. It is up to each country to determine how high the rate of limit value exceedances may be, and whether this rate applies to all skin types or should be dependent on skin type. For Germany, this quota was set at 0.4 (corresponds to 40% of the number of days), so that special measures must be taken for light skin types from a legal point of view [56]. If the same standards were applied in Colombia, then special measures would even be indicated for road construction workers with darker skin types, as the rate of limit value exceedance days is significantly higher.

Against the background of the clearly different irradiations in Germany and Colombia, it is interesting to take a look at the incidences of NMSC in connection with the skin type according to Fitzpatrick. Since the two nations only seem to differ slightly in the distribution of skin types, but there is significantly higher irradiation in Colombia, there should be a significantly higher incidence of NMSC there. According to current figures, this cannot be confirmed: In Germany, the incidence in men is 186/100,000 citizens and in women it is 143/100,000 citizens [57]. In Colombia, on the other hand, it is about 40/100,000 citizens [58]. However, these figures are only comparable to a limited extent, as in many countries there is a clear underreporting of the figures. Germany is known to report many cases, as there is an incentive system for doctors to do so, especially in a professional context.

Road construction workers as a whole can be classified as a cohort of highly irradiated workers.

### 4.2. Prevention Concepts

The prevention requirements to protect the outdoor workers from skin damage caused by solar UVR need to be based on different preventive actions, including technical risk interventions, proper personal skin protection and changes in individual behaviours. However, the lack of information is one of the greatest risks in the workplace, as well as the lack of legal standards and legislation for the prevention of this occupational risk.

The effectiveness of targeted prevention measures should be relevant, and some preventive and early interventions could be applied to reduce the impact of solar UV risks.

A strong recommendation should be to avoid solar exposure irradiation between 10:00 a.m. and 3:00 p.m. to avoid the daily maximum, to try to take breaks in the shade if possible, including breaks in UV-shielded areas during work and lunch time, as this will reduce the risk of possible skin damage. Also, it is relevant to promote shift work schedules in order to avoid outdoor work during the highest UVR exposure.

Since the results of the study are classified as high risk, all control measures and delivery of all personal protection elements (PPE) to exposed workers must be implemented to minimize this risk. It is important to keep the most vulnerable areas of the skin covered (for example, back of the neck, head and arms).

Among these control methods is the recommendation to wear caps or hats with a wide brim (minimum 7 cm, ideal 10 cm) that covers the face and head, which are the areas that are most exposed to sunlight, and also to use a helmet and hanging flap. Sunglasses should meet the standard requirements of shape (e.g., size of the glasses, close fitting) and filtering power of the glasses. It is necessary to wear dark coloured textile-polyester fiber clothing (dark green, dark grey and blue), which is made of permeable material to avoid thermal overload, and appropriate clothing should include long-sleeved shirts. It is also recommended to use waterproof sunscreen (recommended SPF 50+) which adds useful protection to parts of the body that aren’t easy to protect.

It is also important that employers carry out UVR risk management by adopting certain measures, and for that reason it is crucial to inform workers about the specific risks and possible harmful effects of UVR exposure, mainly on the eyes and skin. Awareness raising campaigns and prevention initiatives addressing outdoor workers should be relevant.

Furthermore, the self examination and health surveillance for outdoor workers is essential, and the identification of exposed workers in the activities or tasks in order to detect the jobs that require additional protection measures or barriers that allow the elimination or minimization of the risk is important.

Specialised areas in the companies should track the pre-employment and periodical medical examinations of outdoor workers to avoid the damage effect due to solar UVR exposure. Furthermore, reporting any diagnosed diseases could help to develop a better prevention strategy.

Germany has enshrined occupational health prevention (OHP) in law, and this could serve as a proposal for the international community [55]. OHP must be offered to every worker in Germany whose job meets certain criteria. With regard to UVR exposure, these include the assessment of the exposure period in the months of April to September and the daily period from 11:00 a.m. to 4:00 p.m. (CEST). If a worker has worked outdoors for more than one hour on more than 50 days during this period, they must be offered an OHP. Thus, the German Federal Ministry of Labour and Social Affairs has established a definition of outdoor workers at risk from solar UVR. This legislation is supported by extensive measurements of actual UV exposure of workers in Germany [59]. Recently published research has shown the impact of the German legislation on the number of workers affected and what this would mean for the whole world [46]. It can be assumed that more than half a billion employees worldwide are in need of occupational health prevention, probably significantly more if the findings in this study on constant high irradiation in tropical environments are taken into account. Road construction workers also belong to the group of workers who must benefit from occupational health prevention.

### 4.3. Strenghts and Limitations

This study has limitations. Even if the test persons are well monitored during the long duration of the measurement campaign and are revisited frequently, incorrect wearing of the dosimeter cannot be ruled out in some cases. In principle, the wearing of the dosimeter can be detected by checking the acceleration sensor, but not the correct attachment to the left upper arm. Experience shows, however, that this error occurs only in extremely rare cases. Long-term measurements in particular help to minimize this effect. Furthermore, it must be taken into account that every employee behaves differently from others in parts of their work. Accordingly, the study of an occupational profile is always associated with a certain degree of scatter. By carefully selecting the test persons and their activities, this effect could be minimised. The derivation of findings from the raw data can also be associated with uncertainties. Whenever extrapolation is done, assumptions have to be made (for example, the number of working days per month or per year) and detailed information is lost (this is the nature of averages). Where there is an interpreted range, we have discussed this in the manuscript.

The strength of this study lies in the large number of usable measurements, as well as the resulting statistical accuracy. Furthermore, it has been possible to study similar occupational profiles in both countries and to obtain information on both behavioral and exposure scenarios through direct comparison. This makes it possible in particular to study the influence of the different climatic zones. Especially the resolution on half-hourly level allows the identification of phases of increased risk and thus the derivation of preventive measures. With regard to the technical procedure of the measurement campaigns, it is of particular advantage that we were able to use theidentical measurement technology for all measurement locations and that these corresponded were state of the art. In addition, our measurements were robust against a possible decrease in compliance of the test persons, as this did not lead to a reduction in data quality, but at most could have led to a decrease in the amount of data.

## 5. Conclusions

Our work focused on studying the influence of the climate zone on both irradiation and its course using the example of an occupational group of road construction workers. The basic expectation derived from the scientifically known path of the sun over the climate zones was confirmed. However, what this means for personal irradiation was unknown until now and can be clarified.

Many studies to determine UV irradiation have only used short time periods for measurement [43,44,60,61,62], often without considering whether the findings can be extrapolated to annual irradiation. According to our results, it seems sufficient within the tropical zone to measure irradiation at selected time points with common weather conditions, as extrapolation to the whole year is easier here. The situation is different in temperate latitudes, where the annual course of UVR is also superimposed on apparently changing personal behaviour. As a result, people seek direct sunlight more often in spring when the weather becomes nicer than in summer when it is also very hot.

Experience in occupational health and safety has shown that the implementation of measures is often only effective if there are legally prescribed exposure limits. However, this is not yet the case, either in Europe or in other regions of the world. In Europe, there are only minimum regulations for protection against UVR from artificial sources, which also contain exposure limit values [63]. If one converts the thresholds prescribed there to the exposure from solar UVR, then an exposure limit value of 1 SED would have to be used. Against this background, this paper is also intended to serve as a petition for the introduction of a limit value concept. The International Commission on Non-Ionising Radiation Protection (ICNIRP) also took a scientific position on this some time ago [64].

Future prevention concepts must not be limited to the introduction or ordering of pure measures. In an increasingly complex information society, everyone wants to understand why a measure is necessary (informed consent). The description of an abstract danger that also lies in the future—such as skin cancer caused by UVR—is now no longer sufficient. For the topic of UVR, this means that the UV exposure values scientifically determined in this and the other papers must be communicated simply and ideally should be displayed by directly indicating measuring instruments, for example at construction sites. The UV index, which can be currently measured or indicated in the weather forecast, lends itself to this [65]. The direct connection between the static measurements carried out on a horizontal plane and the measurements on the person is not simple, but can be established by approximations [33,34].

The integration of digital tools into prevention work can be of great benefit. Websites or apps already exist today that deal with protection from solar UVR. This is not only about generally valid information, but also about (daily) current information about the weather, the expected UV irradiation, indicated by the UV index, as well as about appropriate protective measures. Such websites or apps can be found by searching in the corresponding portals; no product is to be specifically advertised here.

It is precisely from the distribution of UV irradiation throughout the day that we could learn that the siesta, which has always been incorporated into the culture in countries of the tropics, is of great importance in prevention. This leads to a considerable reduction in daily UVR exposure and presumably also to the avoidance of excessive heat. Due to the increased irradiation of carcinogenic UV-B radiation in temperate latitudes as a result of ozone depletion [66,67,68], it would be advisable for organisational measures to make use of this knowledge from the tropics. The fact that people in temperate latitudes are disproportionately exposed in spring underlines that the danger from UVR is widely underestimated.

Behavioural and situational prevention can benefit equally from the findings of this work. Prevention work should follow several paths:

1. Development of appropriate technical protective measures: The maximum possible level of protection can be used, adapted to the place of work. The less radiation that reaches the workers, the lower the risk;
2. Implementation of suitable organisational protective measures: Adjustment of working hours, introduction of adequate occupational health measures, instruction and training at regular intervals;
3. Development and introduction of personal protective measures: Not every item of clothing necessarily has to be certified. Often normal street clothes are sufficient. It is important that they cover the body. Particularly in the case of personal protective measures, such as clothing, care should be taken to ensure that they are designed to provide the required level of protection and that they do not promote other hazards, such as overheating.

Non-melanocytic skin cancer caused by UVR is preventable through adequate sun-safe behaviour. This makes it a prime example of a holistic approach to prevention, the goal of which must be to prevent all skin cancers. However, this lofty goal only has a chance of success if all those concerned work together, including training companies, as well as employers, employees, medical societies and insurers.

Ideally, such efforts are international, as workers in all countries are exposed to solar UVR. While cultural differences can sometimes be a difficulty in creating a universal set of rules across borders, they also present many opportunities. One can learn from people in other countries, because a cultural development often includes prevention that has grown over centuries. This work also demonstrates this.

## Figures and Tables

**Table 1:** Daily mean values for German and Colombian road construction workers, respectively. The daily mean values for every month (Ø) are shown with their respective standard error (Err). All values are given in J/m^2^.

	Jan.	Feb.	Mar.	Apr.	May	Jun.	Jul.	Aug.	Sep.	Oct.	Nov.	Dec.
	**Germany**											
Ø	-	-	-	361.1	329.3	365.1	402.1	282.3	172.5	85.6	-	-
Err	-	-	-	19.7	15.7	14.6	18.0	17.5	9.5	6.7	-	-
	**Colombia**											
Ø	479.5	723.3	889.8	-	-	-	-	686.7	798.4	519.1	586.0	766.9
Err	54.2	70.9	77.5	-	-	-	-	63.4	139.1	53.7	47.3	75.4

## Data Availability

Data displayed in graphs can be accessed by contacting the corresponding author.
